# Supplementary material for: IgCaller for reconstructing immunoglobulin gene rearrangements and oncogenic translocations from whole-genome sequencing in lymphoid neoplasms
Source: Nat Commun. 2020 Jul 7;11:3390. doi: 10.1038/s41467-020-17095-7 (PMC7341758; doi:10.1038/s41467-020-17095-7)
Supplement: Supplementary file 1 — Supplementary Information [file 41467_2020_17095_MOESM1_ESM.pdf]

IgCaller for Reconstructing Immunoglobulin Gene Rearrangements and Oncogenic Translocations from Whole-Genome Sequencing in Lymphoid Neoplasms

Nadeu et al.

Supplementary Information

Supplementary Figures

a

MCL: 1412 (SSeq)

**Result summary:**	**Productive IGH rearranged sequence** (no stop codon and in-frame junction)		
V-GENE and allele	Homsap IGHV4-39*01 F	score = 1432	identity = 99.31% (289/291 nt)
J-GENE and allele	Homsap IGHJ3*01 F (a)	score = 160	identity = 80.00% (40/50 nt)
D-GENE and allele by IMGT/JunctionAnalysis	Homsap IGHD3-3*01 F	D-REGION is in reading frame 3	
FR-IMGT lengths, CDR-IMGT lengths and AA JUNCTION	[25.17.38.11]	[10.7.13]	CARLVFGVVIGLDVW

(a) Other possibilities: Homsap_IGHJ6*02 (highest number of consecutive identical nucleotides)

Closest J-REGIONs

	Score	Identity
Homsap IGHJ3*01 F	160	80.00% (40/50 nt)
Homsap IGHJ3*02 F	151	78.00% (39/50 nt)
Homsap IGHJ6*02 F	139	69.35% (43/62 nt)
Homsap IGHJ5*01 F	129	72.55% (37/51 nt)
Homsap IGHJ6*01 F	126	66.67% (42/63 nt)

b

MCL: 1412 (WGS)

chr14 (hg19)

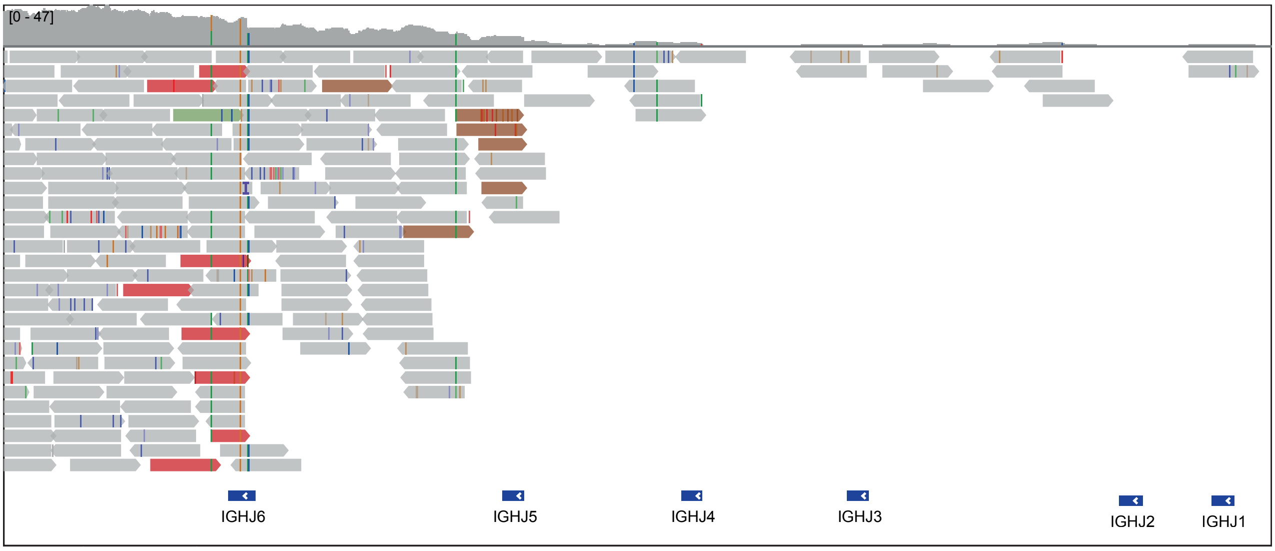

c

C1-CLL: 60

Sanger sequencing:

**Result summary:**	**Productive IGH rearranged sequence** (no stop codon and in-frame junction)		
V-GENE and allele	Homsap IGHV3-7*03 F	score = 1075	identity = 86.11% (248/288 nt)
J-GENE and allele	Homsap IGHJ4*03 F (a)	score = 168	identity = 83.33% (40/48 nt)
D-GENE and allele by IMGT/JunctionAnalysis	Homsap IGHD3-3*01 F	D-REGION is in reading frame 2	
FR-IMGT lengths, CDR-IMGT lengths and AA JUNCTION	[25.17.38.11]	[8.8.15]	CVRENEFWSGGWGLDGW

(a) Other possibilities: Homsap_IGHJ6*02 (highest number of consecutive identical nucleotides)

Closest J-REGIONs

	Score	Identity
Homsap IGHJ4*03 F	168	83.33% (40/48 nt)
Homsap IGHJ6*02 F	166	74.19% (46/62 nt)
Homsap IGHJ6*01 F	162	73.02% (46/63 nt)
Homsap IGHJ6*04 F	162	73.02% (46/63 nt)
Homsap IGHJ4*01 F	150	79.17% (38/48 nt)

d

C1-CLL: 60 (WGS)

chr14 (hg38)

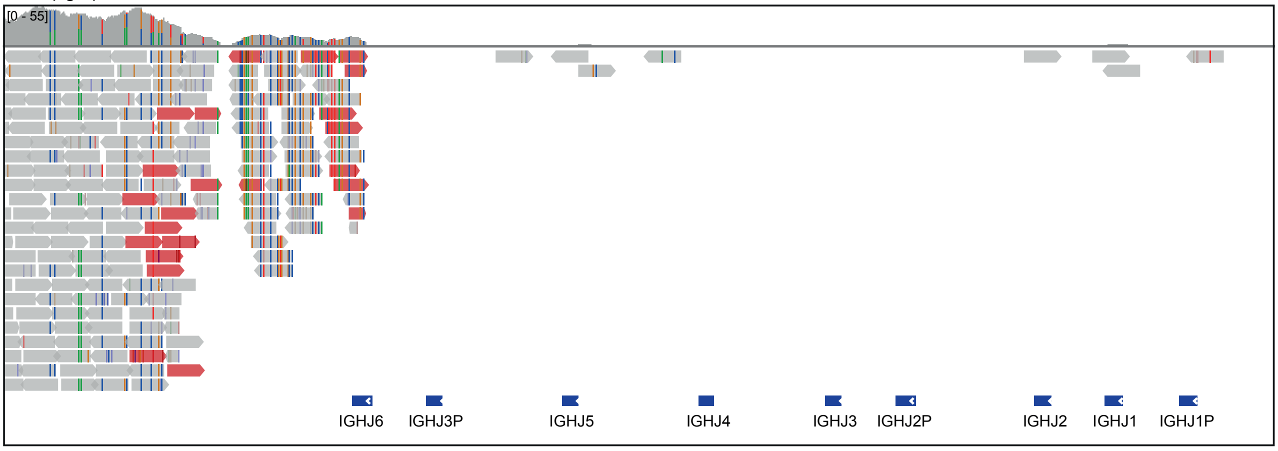

Supplementary Figure 1. Discrepancies between IMGT/V-QUEST and IgCaller. a, IMGT/V-QUEST result from SSeq of MCL case 1412. Multiple possible J genes are reported. **b,** Integrative Genomics Viewer (IGV) image showing the WGS reads aligning within the IGHJ locus. Bar plots on top show the coverage in each position. Each horizontal bar represents a WGS read. Gray positions represent wild-type bases both in the coverage bar plot and reads. Colored positions represent mutations. Abnormal insert size reads spanning the IGHJ6-GHV4-39 rearrangement identified by IgCaller are depicted in red. No reads supporting a potential IGHJ3 rearrangement are found at WGS level. Reads spanning the t(11;14) (IG/*CCND1*) found by IgCaller in this MCL sample are labelled in brown. **c, d,** Same as a, b, respectively, but for the rearrangements observed by SSeq and WGS in C1-CLL case 60. Red-labeled reads mapping downstream of IGHJ6 on panel 'd' correspond to a deletion on the non-V(D)J rearranged allele with a second breakpoint found between the IGHD6-19 and IGHD5-18 genes.

a

C1-CLL: 134 (SSeq)

**Result summary:**	**Productive IGH rearranged sequence** (no stop codon and in-frame junction)		
V-GENE and allele	Homsap IGHV5-10-1*03 F	score = 1435	identity = **100.00%** (288/288 nt)
J-GENE and allele	Homsap IGHJ4*02 F	score = 192	identity = 95.24% (40/42 nt)
D-GENE and allele by IMGT/JunctionAnalysis	Homsap IGHD6-19*01 F	D-REGION is in reading frame 3	
FR-IMGT lengths, CDR-IMGT lengths and AA JUNCTION	[25.17.38.9]	[8.8.14]	CARLQSLGLTNPFDYW

b

C1-CLL: 134 (WGS)

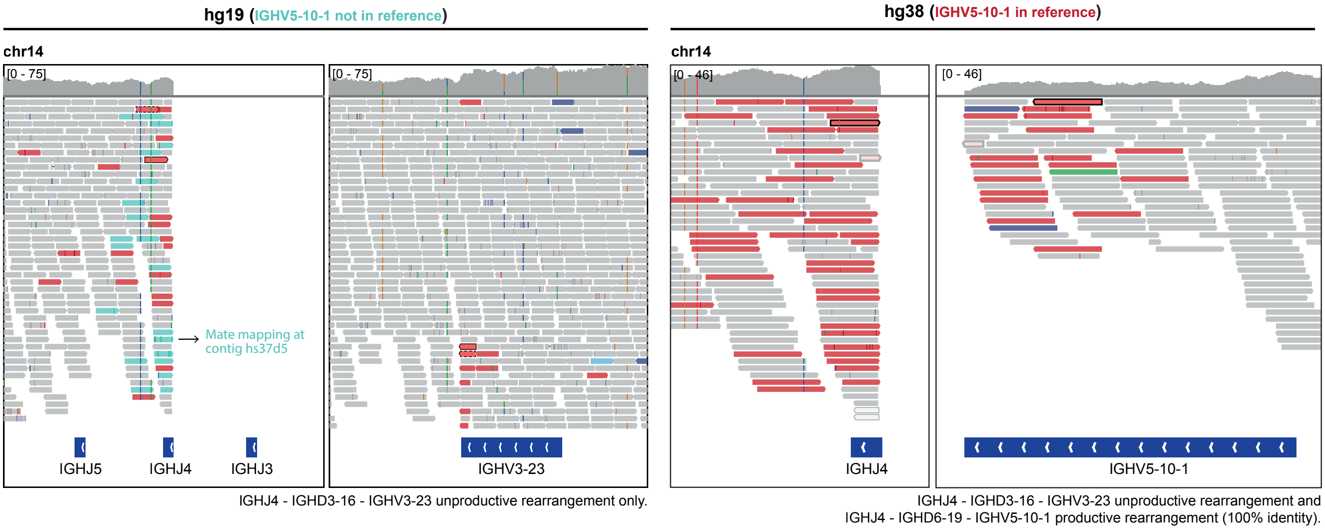

Supplementary Figure 2. Reference-based incongruent results of IgCaller. a, IMGT/V-QUEST result for CLL case 134 based on SSeq. **b,** WGS reads aligning to hg19 reference genome (left). Only one unproductive rearrangement is identified by IgCaller, while the productive rearrangement involving the IGHV5-10-1 is not called due to the fact that the IGHV5-10-1 gene is not present in the hg19 reference genome used, and IGHV5-10-1 reads mapped to contig hs37d5. When the WGS data is aligned to the hg38 reference genome (right), which includes the IGHV5-10-1 gene, the productive rearrangement identified by SSeq is also detected by IgCaller.

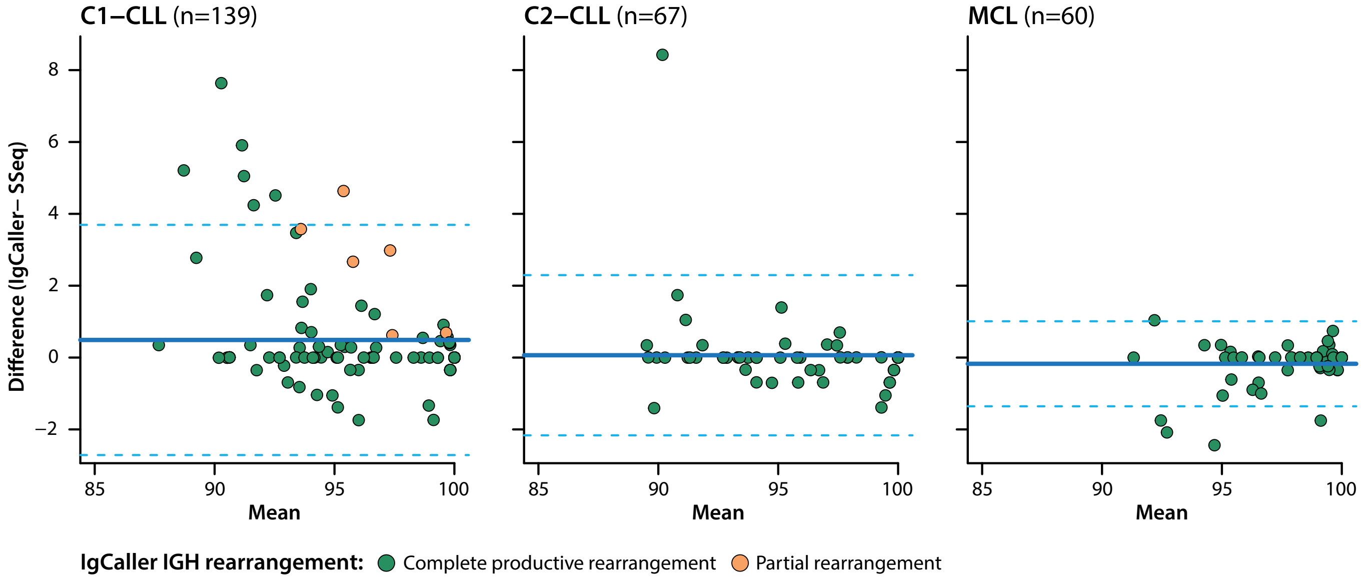

Supplementary Figure 3. Extended benchmark of IgCaller. Bland-Altman plots of the comparison of the percentage of identity of the rearranged IGHV sequence identified by IgCaller and SSeq/NGS. Solid, dark blue line represents the mean difference, while dashed, light blue lines correspond to the 95% limits of agreement (average distance ±1.96 standard deviation of the difference). Source data are provided as a Source Data file.

C1-CLL: 60

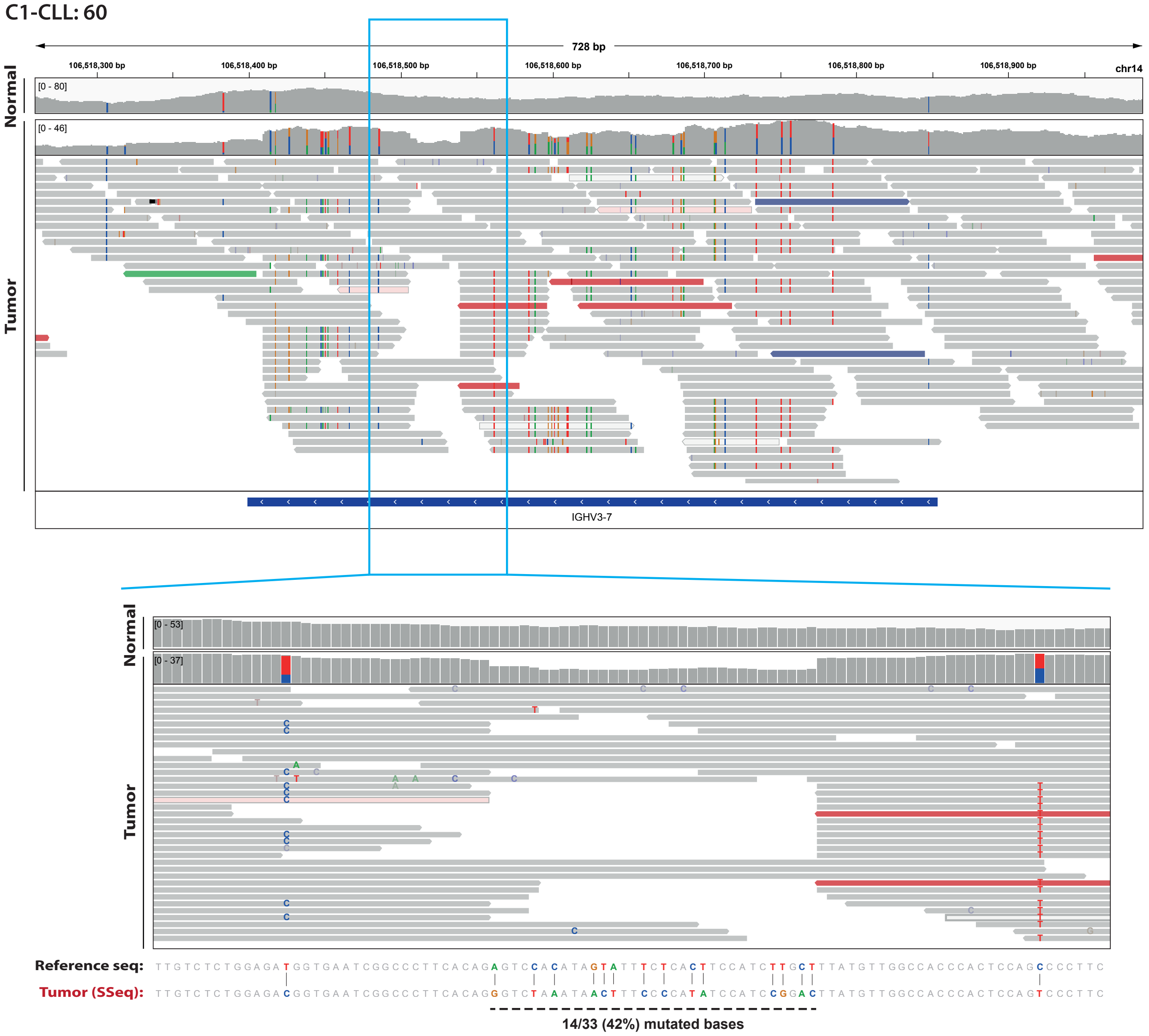

Supplementary Figure 4. High density of somatic mutations impairs the alignment of WGS reads. Representation of the WGS reads of CLL 60 aligning to the IGHV3-7 gene involved in the productive V(D)J rearrangement identified both by SSeq and WGS. The region highlighted in blue shows a drop of coverage and the lack of somatic mutations. By SSeq (bottom) we observed a high density of somatic mutations within the 33bp window of low coverage, suggesting that WGS reads carrying these mutations do not align to this region due to this cluster of mutations.

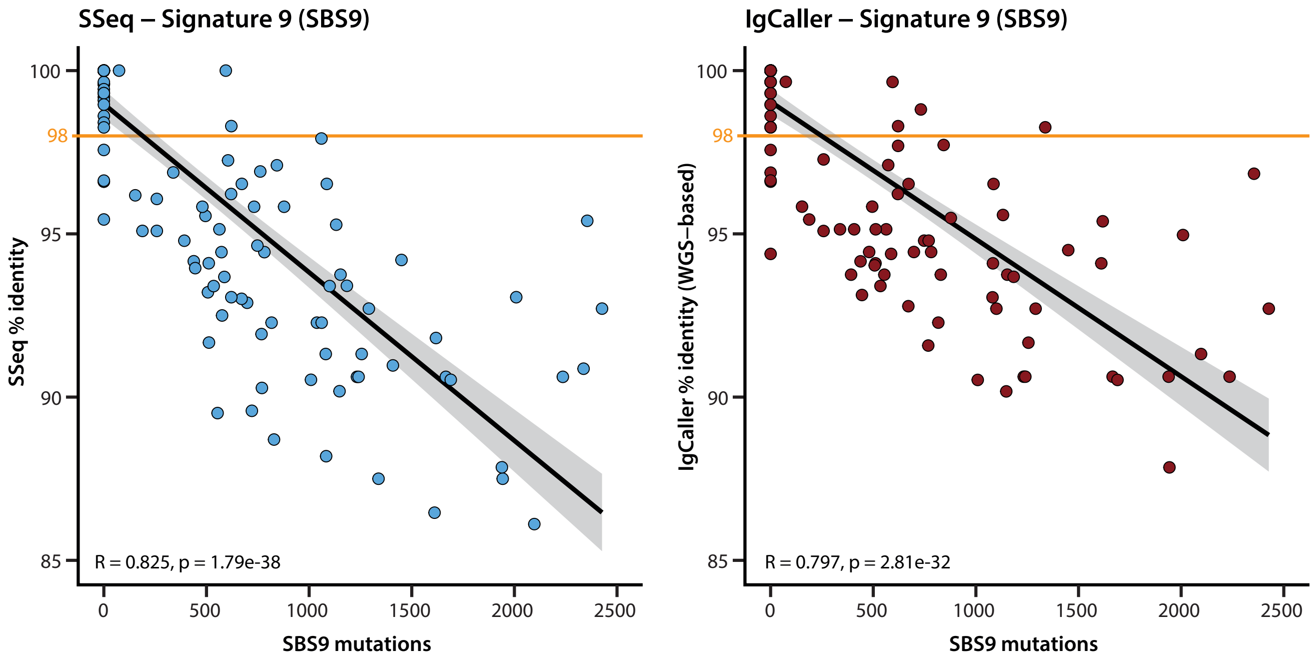

Supplementary Figure 5. Signature 9 mutations vs. identity of the rearranged IGHV gene in the C1-CLL cohort. Correlation between the number of mutations associated to signature 9 (SBS9) and the identity of the rearranged sequence observed by SSeq (left) and IgCaller (right). *P* values are from t-test. The gray area represents 95% confidence intervals. N=152 CLL patients from C1-CLL cohort. Source data are provided as a Source Data file.

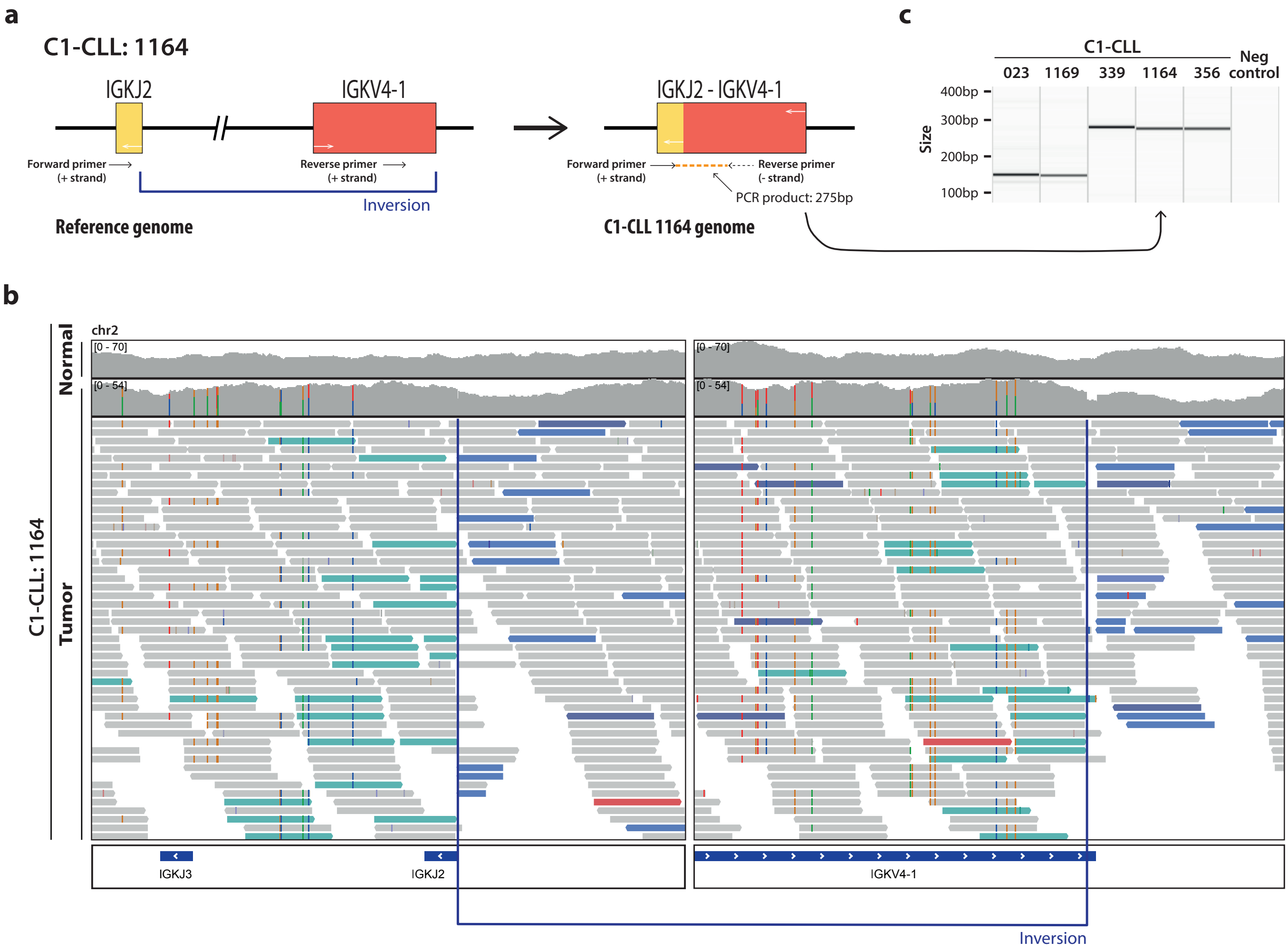

Supplementary Figure 6. Verification of IGK-inversion rearrangements. a, Schema of the IGK rearrangement mediated by an inversion of the IGKV gene found by IgCaller in C1-CLL 1164. White arrows represent the coding strand. PCR primers used in the verification are shown in black arrows. **b,** IGV image of the WGS reads of C1-CLL 1164 case aligning to the IGKJ2-IGKV4-1 rearrangement identified. Reads supporting the inversion leading to a productive rearrangement are depicted in turquoise. Dark blue reads show the recursive inversion supporting the second new junction of the inversion. **c,** PCR products of the 5 IGK-inversion rearrangements verified by PCR and SSeq. PCR and SSeq was performed once for each sample. All samples derive from the same experiment and gels were processed in parallel. The uncropped gel image is provided as a Source Data file.

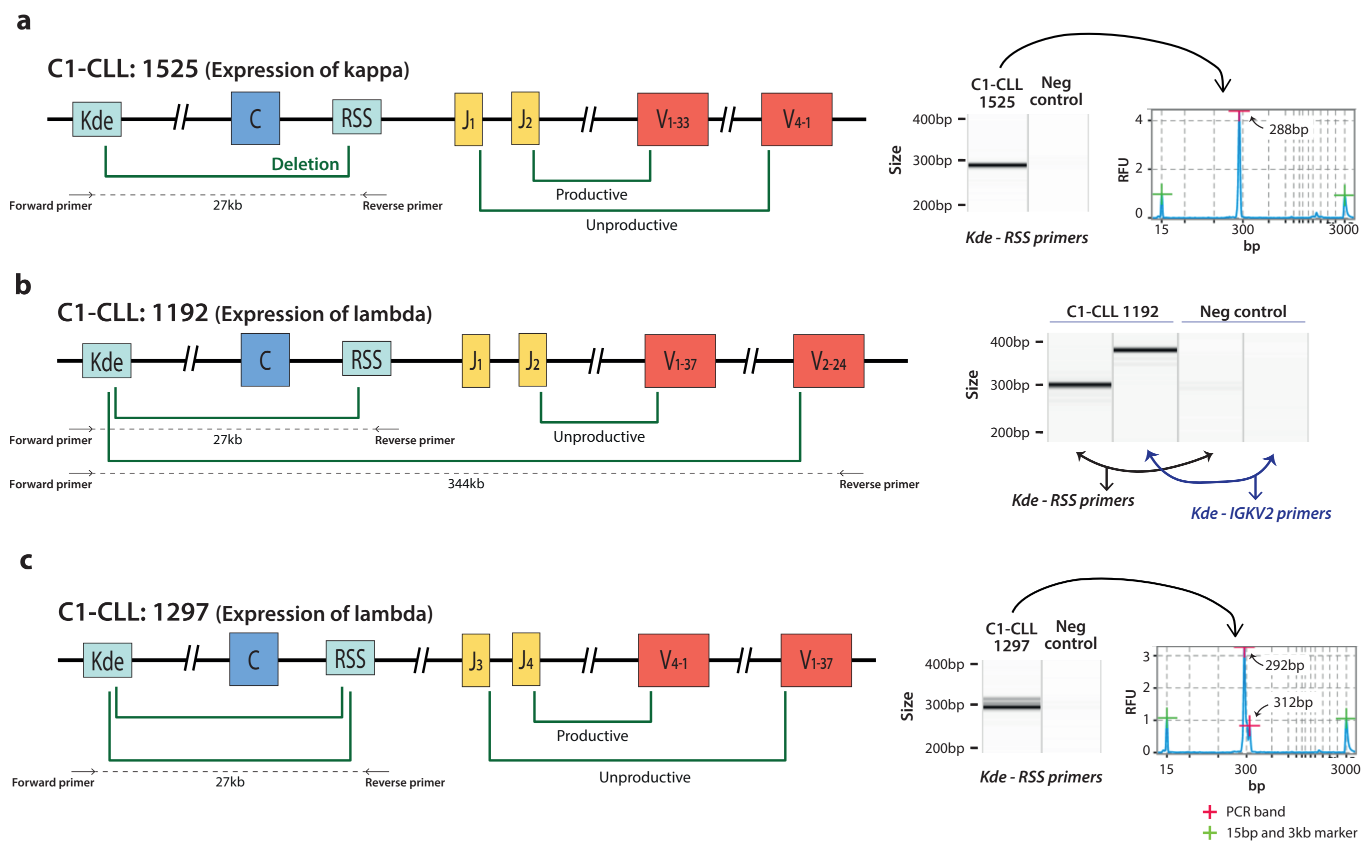

Supplementary Figure 7. IGK Kde and RSS deletions verified by PCR. a, Schema of the IGK rearrangements found in the IGK locus using IgCaller in CLL patient 1525 (left). PCR verification of the deletion within the kappa deleting element (Kde) and the intron recombination signal sequence (RSS, right). **b,** IGK rearrangements found by IgCaller (left) together with the PCR verification of both Kde-RSS and Kde-IGKV2-24 deletions detected in CLL patient 1192. **c,** Representation of one case carrying two independent Kde-RSS deletions which eliminate one productive and one unproductive rearrangement (left). PCR shows the presence of two Kde-RSS products confirming the presence of two Kde-RSS deletions (right). Each PCR experiment was performed once. All samples derive from the same experiment and gels were processed in parallel. The uncropped gel images are provided as a Source Data file.

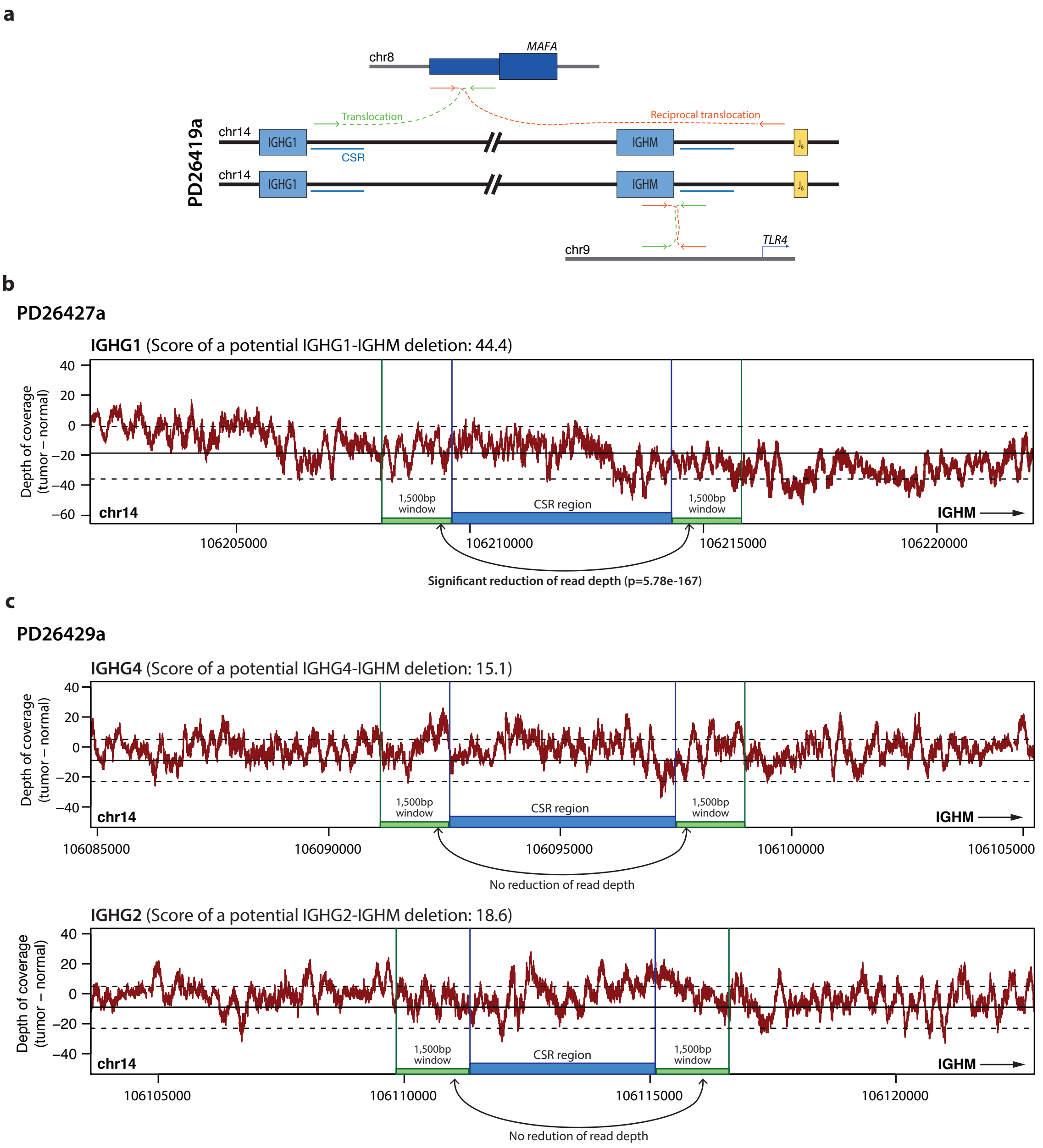

Supplementary Figure 8. Potentially discordant CSR in three MM cases. a, Cartoon of the two secondary IGH translocations observed in MM case PD26419a. These two translocations might cause the loss of the IGH constant region of both alleles, and therefore we only observed IGLC expression by FC. In agreement, IgCaller did not identified any CSR in this case as well as it identified both translocations. **b,** Case PD26427a expressed IGLC by FC. IgCaller identified a CSR involving IGHG1. We have not observed secondary IGH translocations that could explain the loss of IGH expression. **c,** Case PD26429a expressed IgG by FC. IgCaller identified abnormal insert sizes reads that could support the presence of an IGHG4-IGHM and/or IGHG2-IGHM deletion. However, a significant reduction of the read depth before and after the CSR of IGHG4 and IGHG2, which is mandatory in order to be called as CSR by IgCaller, is not detected. Thus, this case was classified as IGHM (i.e. no CSR found). *P* values are from Wilcoxon test.

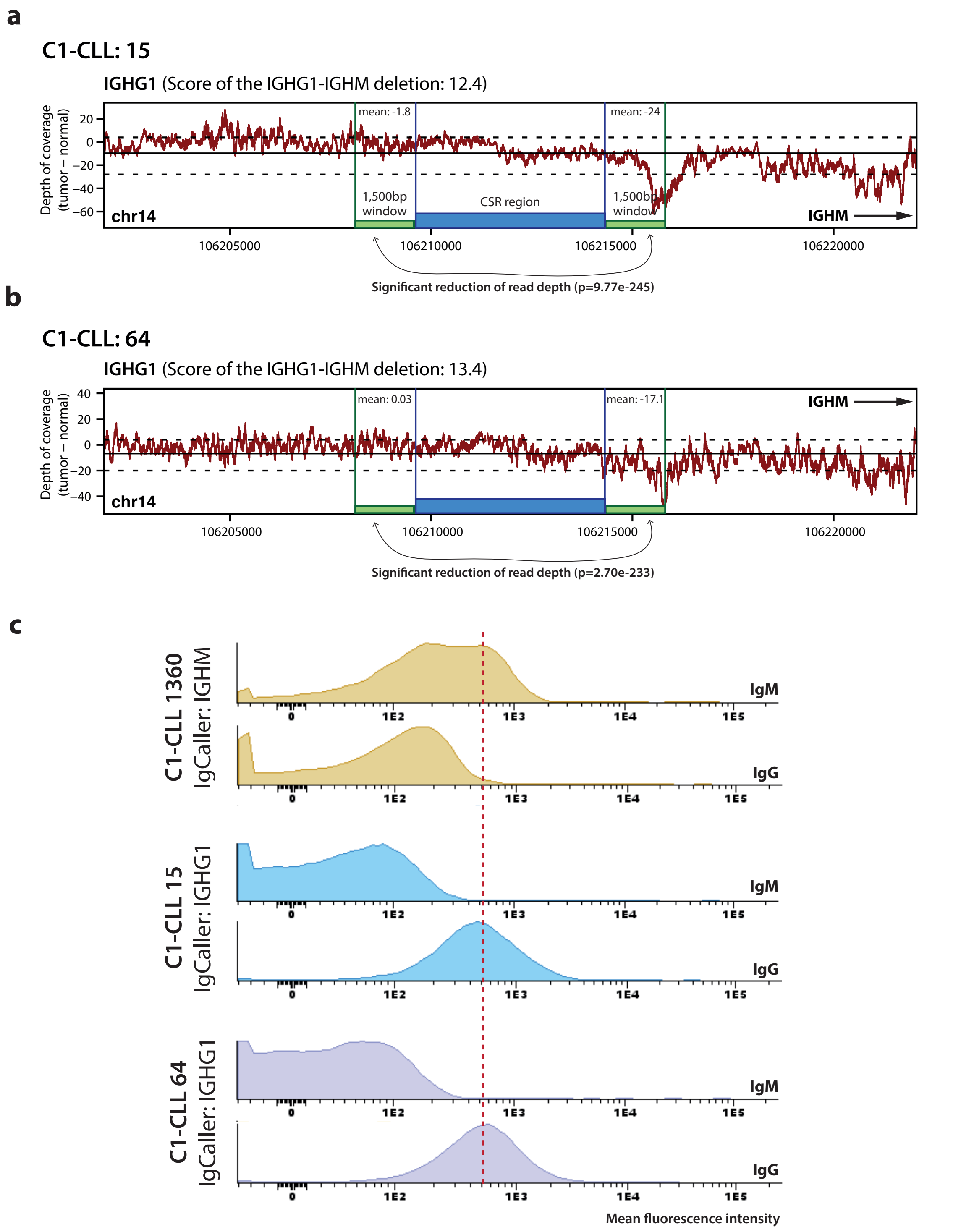

Supplementary Figure 9. CSR verification in CLL cases. a, b, Representation of the WGS IGHG1 region of two cases with CSR involving IGHG1 as identified by IgCaller. *P* values are from Wilcoxon test. **c,** FC analyses of these two cases confirmed the expression of IgG. One CLL case with no CSR identified by IgCaller is also shown. In this latter case, the FC analysis also confirmed the IgM expression identified by IgCaller.

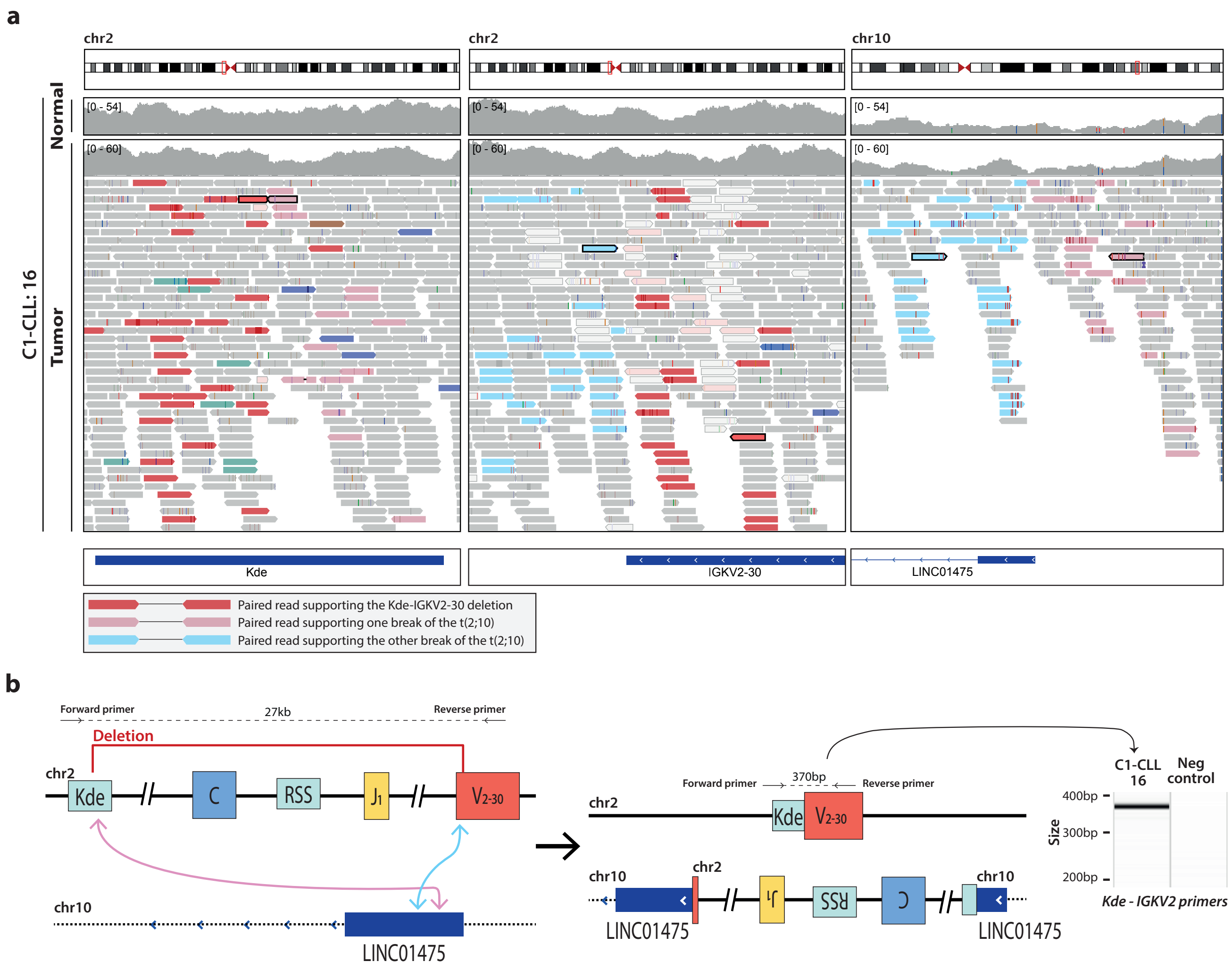

Supplementary Figure 10. IGK insertion in LINC01475 identified by IgCaller. a, IGV image of the break points of the translocations in which the IGK Kde-IGKV2-2 regions is inserted in LINC01475. Reads supporting each specific rearrangement are colored**. b,** Cartoon of the rearrangement observed (left), and PCR verification of the Kde-IGKV2-2 deletion in chromosome 2 (right). PCR experiment was performed once. All samples derive from the same experiment and gels were processed in parallel. The uncropped gel image is provided as a Source Data file.

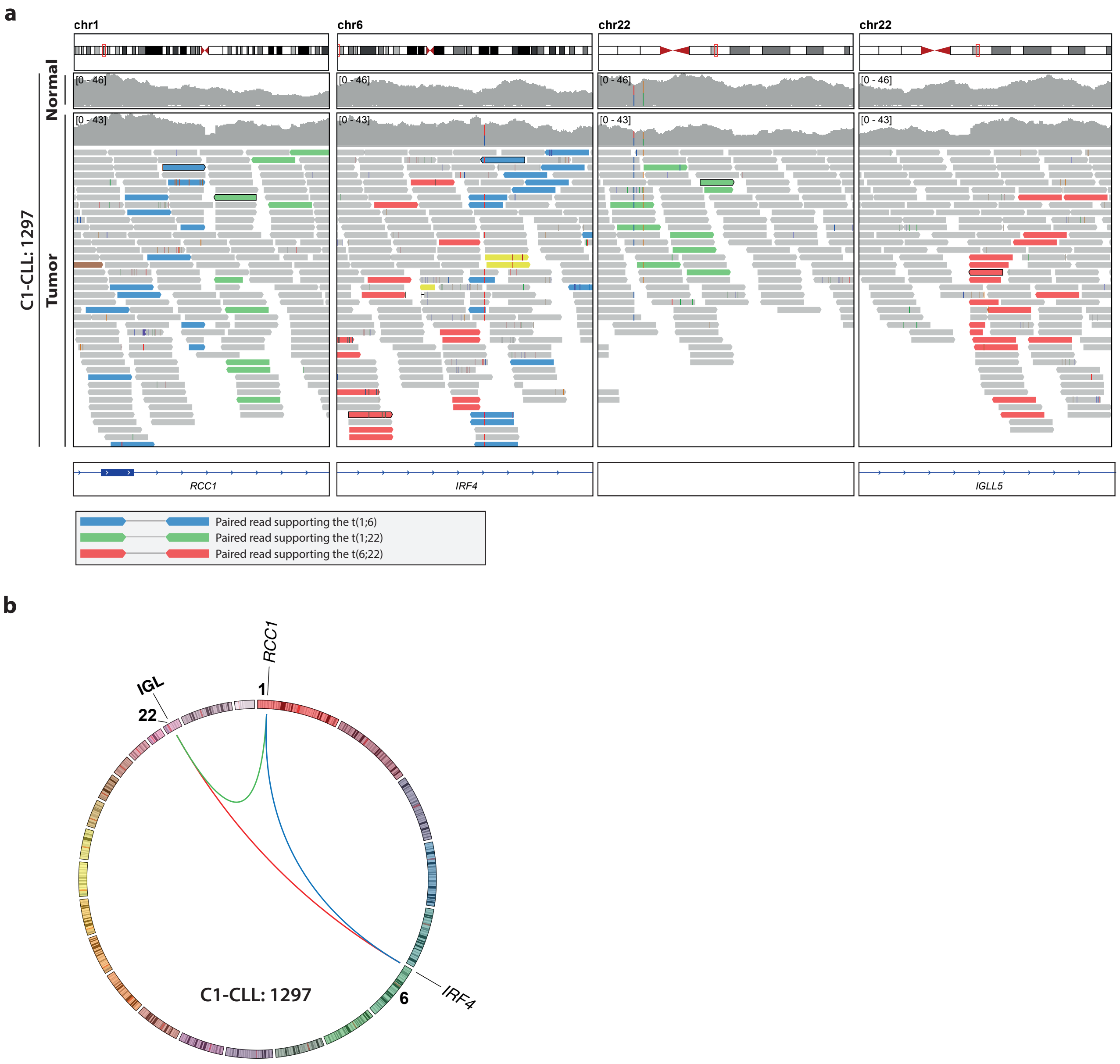

Supplementary Figure 11. ***IRF4*-IGL and *RCC1*-IGL translocations identified by IgCaller.** **a,** IGV image of the break points of the translocations observed. Reads supporting each specific rearrangement are colored. Note that the t(1;6) [*RCC1-IRF4*] was not detected by IgCaller due to the fact that the program focuses only in characterizing rearrangements involving the Ig loci. **b,** Cartoon of the rearrangement observed.

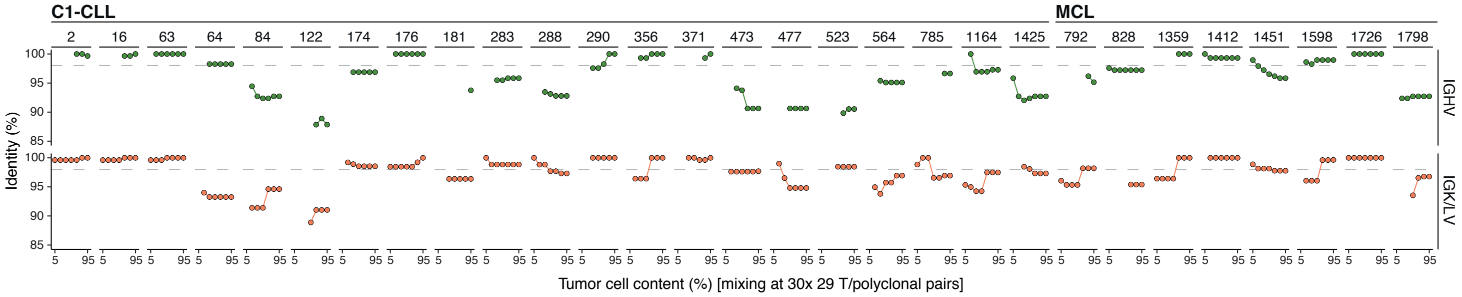

Supplementary Figure 12. Ig gene identity at distinct polyclonal contamination rates. V gene identity for IGH (*top*) and IGK/L (*bottom*) gene rearrangement for each case at different tumor cell contents when the clonal tumor population is mixed with a polyclonal-like population. Source data are provided as a Source Data file.
