# Supplementary material for: IgCaller for reconstructing immunoglobulin gene rearrangements and oncogenic translocations from whole-genome sequencing in lymphoid neoplasms
Source: Nat Commun. 2020 Jul 7;11:3390. doi: 10.1038/s41467-020-17095-7 (PMC7341758; doi:10.1038/s41467-020-17095-7)
Supplement: Supplementary file 3 — Reporting Summary [file 41467_2020_17095_MOESM3_ESM.pdf]

natureresearch

Corresponding author(s): Ferran Nadeu

Last updated by author(s): Jun 4, 2020

Reporting Summary

Nature Research wishes to improve the reproducibility of the work that we publish. This form provides structure for consistency and transparency in reporting. For further information on Nature Research policies, see Authors & Referees and the Editorial Policy Checklist .

Statistics

For all statistical analyses, confirm that the following items are present in the figure legend, table legend, main text, or Methods section.

n/a	Confirmed	
☐	☒	The exact sample size (*n*) for each experimental group/condition, given as a discrete number and unit of measurement
☐	☒	A statement on whether measurements were taken from distinct samples or whether the same sample was measured repeatedly
☐	☒	The statistical test(s) used AND whether they are one- or two-sided<br>*Only common tests should be described solely by name; describe more complex techniques in the Methods section.*
☐	☒	A description of all covariates tested
☐	☒	A description of any assumptions or corrections, such as tests of normality and adjustment for multiple comparisons
☐	☒	A full description of the statistical parameters including central tendency (e.g. means) or other basic estimates (e.g. regression coefficient) AND variation (e.g. standard deviation) or associated estimates of uncertainty (e.g. confidence intervals)
☐	☒	For null hypothesis testing, the test statistic (e.g. *F*, *t*, *r*) with confidence intervals, effect sizes, degrees of freedom and *P* value noted<br>*Give P values as exact values whenever suitable.*
☒	☐	For Bayesian analysis, information on the choice of priors and Markov chain Monte Carlo settings
☒	☐	For hierarchical and complex designs, identification of the appropriate level for tests and full reporting of outcomes
☐	☒	Estimates of effect sizes (e.g. Cohen's *d*, Pearson's *r*), indicating how they were calculated

Our web collection on statistics for biologists contains articles on many of the points above.

Software and code

Policy information about availability of computer code

Data collection	Publicly available software was used for whole-genome sequencing data processing: BWA-MEM (v0.7.15 and v0.7.17), samtools (version 1.6 and 1.9), biobambam2 (v2.0.65).
Data analysis	IgCaller (new software released with this paper; https://github.com/ferrannadeu/IgCaller), R (v3.5.0), LymphoTrack MiSeq Data Analysis (v2.3.1), MiXCR (v3.0.12).

For manuscripts utilizing custom algorithms or software that are central to the research but not yet described in published literature, software must be made available to editors/reviewers. We strongly encourage code deposition in a community repository (e.g. GitHub). See the Nature Research guidelines for submitting code & software for further information.

Data

Policy information about availability of data

All manuscripts must include a data availability statement. This statement should provide the following information, where applicable:

- Accession codes, unique identifiers, or web links for publicly available datasets
- A list of figures that have associated raw data
- A description of any restrictions on data availability

Sequencing data of the C1-CLL cohort, four MCL samples, MM, and DLBCL is available from the European Genome-phenome Archive (EGA) under accession numbers EGAS00001001306, EGAS00001000510, EGAS00001001299, and EGAS00001002936, respectively. Previously unpublished data has been deposited at EGA under accession numbers EGAS00001004165 (for tumor/normal WGS of 57 MCL cases) and EGAS00001004298 (IG reads for C2-CLL and B-NHL cohorts as well as 3 MCL cases). All other data are included in the supplemental information or available from the authors upon reasonable requests.

Field-specific reporting

Please select the one below that is the best fit for your research. If you are not sure, read the appropriate sections before making your selection.

- [x] Life sciences
- [ ] Behavioural & social sciences
- [ ] Ecological, evolutionary & environmental sciences

For a reference copy of the document with all sections, see nature.com/documents/nr-reporting-summary-flat.pdf

Life sciences study design

All studies must disclose on these points even when the disclosure is negative.

Sample size	We use the available WGS data for 404 B-cell neoplasms: 230 cases of CLL in two independent cohorts of 152 (cohort 1 [C1]) and 78 (cohort 2 [C2]), 64 cases of MCL, 30 MM, 73 DLBCL, and 7 mature B-cell non-Hodgkin lymphomas (B-NHL).
Data exclusions	No data exclusions.
Replication	We have not performed experiments where replication was needed. We have used multiple independent cohorts to test our method.
Randomization	No randomization performed because we were not working with experimental groups, but just characterizing a patient-specific gene rearrangment. We have used different independent cohorts of patients to validate our method. We have not studied any condition/effect were randomizaiton was needed.
Blinding	We were not studying any effect/treatment/outcome where blinding was necessary.

Reporting for specific materials, systems and methods

We require information from authors about some types of materials, experimental systems and methods used in many studies. Here, indicate whether each material, system or method listed is relevant to your study. If you are not sure if a list item applies to your research, read the appropriate section before selecting a response.

Materials & experimental systems

n/a	Involved in the study	
x		Antibodies
x		Eukaryotic cell lines
x		Palaeontology
x		Animals and other organisms
x		Human research participants
x		Clinical data

Methods

n/a	Involved in the study	
x		ChIP-seq
x		Flow cytometry
x		MRI-based neuroimaging
