# Supplementary material for: IgCaller for reconstructing immunoglobulin gene rearrangements and oncogenic translocations from whole-genome sequencing in lymphoid neoplasms
Source: Nat Commun. 2020 Jul 7;11:3390. doi: 10.1038/s41467-020-17095-7 (PMC7341758; doi:10.1038/s41467-020-17095-7)
Supplement: Supplementary file 4 — Description of Additional Supplementary Files [file 41467_2020_17095_MOESM4_ESM.pdf]

Description of Additional Supplementary Files

File Name: Supplementary Data 1-33

Description: This Supplementary Data file includes the following tables:

- Supplementary Data 1. Study cohort.
- Supplementary Data 2. IGH characterization by SSeq and WGS of the C1-CLL cohort.
- Supplementary Data 3. IGH characterization by NGS and WGS of the C2-CLL cohort.
- Supplementary Data 4. IGH characterization by SSeq and WGS of the MCL cohort.
- Supplementary Data 5. IGH characterization by WGS of the MM cohort.
- Supplementary Data 6. IGH characterization by WGS of the DLBCL cohort.
- Supplementary Data 7. IGH characterization by NGS and WGS of the B-NHL cohort.
- Supplementary Data 8. IGH unproductive rearrangements.
- Supplementary Data 9. IGLC characterization by FC and WGS of the C1-CLL cohort.
- Supplementary Data 10. IGLC characterization by FC and WGS of the C2-CLL cohort.
- Supplementary Data 11. IGLC characterization by FC and WGS of the MCL cohort.
- Supplementary Data 12. IGLC characterization by FC and WGS of the MM cohort.
- Supplementary Data 13. IGLC characterization by FC and WGS of the DLBCL cohort.
- Supplementary Data 14. IGLC characterization by WGS of the B-NHL cohort.
- Supplementary Data 15. Verification of IGK-inversion rearrangements.
- Supplementary Data 16. Kde/RSS deletions and unproductive/unexpressed IGLC gene rearrangements.
- Supplementary Data 17. CSR characterization by FC and WGS on the MM cohort.
- Supplementary Data 18. CSR characterization by FC and WGS on the C1-CLL cohort.
- Supplementary Data 19. CSR characterization by WGS on the C2-CLL cohort.
- Supplementary Data 20. CSR characterization by WGS on the MCL cohort.
- Supplementary Data 21. CSR characterization by WGS on the DLBCL cohort.
- Supplementary Data 22. CSR characterization by WGS on the B-NHL cohort.
- Supplementary Data 23. Oncogenic Ig translocations identified by IgCaller.
- Supplementary Data 24. Downsampling analysis.
- Supplementary Data 25. Analysis of normal in tumor contamination at 30x WGS.
- Supplementary Data 26. Analysis of normal in tumor contamination at 60x WGS.
- Supplementary Data 27. Polyclonal contamination analysis.
- Supplementary Data 28. Oligoclonal experiment.
- Supplementary Data 29. Output of MiXCR.
- Supplementary Data 30. Example of the output file containing the high confidence rearrangements identified by IgCaller.
- Supplementary Data 31. Description of the IGH, IGK, and IGL-specific output files.
- Supplementary Data 32. Description of the CSR-specific output file.
- Supplementary Data 33. Description of the oncogenic Ig translocations-specific file.
